# Substance P activates responses correlated with tumour growth in human glioma cell lines bearing tachykinin NK_1_ receptors

**DOI:** 10.1038/sj.bjc.6690039

**Published:** 1999-01

**Authors:** C Palma, F Nardelli, S Manzini, C A Maggi

**Affiliations:** Department of Pharmacology,Via Tito Speri 10, Menarini Ricerche, Pomezia, RM, 00040, Italy; Menarini Ricerche, Via F Rismondo 12 A, Firenze, 50131, Italy

**Keywords:** glioma, substance P, tachykinin NK_1_receptor, cytokine production, proliferation

## Abstract

The neuropeptide substance P (SP), by stimulating tachykinin NK_1_receptors (NK_1_R), triggers a number of biological responses in human glioma cells which are potentially relevant for tumour growth. First, radioligand binding studies demonstrated the presence of tachykinin NK_1_R on SNB-19, DBTRG-05 MG and U373 MG, but not on U138 MG and MOG-G-GCM human glioma cell lines. Second, application of SP or neurokinin A (NKA) to NK_1_R^+^glioma cell lines increased the secretion of interleukin 6 (IL-6) and potentiated IL-6 secretion induced by IL-1β. SP also up-regulated the release of transforming growth factor β1 (TGF-β1) by the U373 MG glioma cell line. Third, SP induced new DNA synthesis and enhanced the proliferation rate of NK_1_R^+^, but not of NK_1_R^−^glioma cell lines. Also, NKA stimulated the proliferation and cytokine secretion in NK_1_R^+^glioma cell lines. All the stimulant effects of SP/NKA on NK_1_R^+^glioma cell lines were completely blocked by a specific tachykinin NK_1_R antagonist, MEN 11467. These data support the potential use of tachykinin NK_1_R antagonist for controlling the proliferative rate of human gliomas. © 1999 Cancer Research Campaign


*British Journal of Cancer* (1999) **79**(2), 236–243
© 1999 Cancer Research Campaign

### Substance P activates responses correlated with tumour growth in human glioma cell lines bearing tachykinin $NK_1$ receptors

**C Palma[1], F Nardelli[1], S Manzini[1] and CA Maggi[2]**

[1]Menarini Ricerche, Department of Pharmacology, Via Tito Speri 10, 00040 Pomezia, RM, Italy; [2]Menarini Ricerche, Via F Rismondo 12 A, 50131 Firenze, Italy

**Summary** The neuropeptide substance P (SP), by stimulating tachykinin $NK_1$ receptors ($NK_1R$), triggers a number of biological responses in human glioma cells which are potentially relevant for tumour growth. First, radioligand binding studies demonstrated the presence of tachykinin $NK_1R$ on SNB-19, DBTRG-05 MG and U373 MG, but not on U138 MG and MOG-G-GCM human glioma cell lines. Second, application of SP or neurokinin A (NKA) to $NK_1R^+$ glioma cell lines increased the secretion of interleukin 6 (IL-6) and potentiated IL-6 secretion induced by IL-1β. SP also up-regulated the release of transforming growth factor β1 (TGF-β1) by the U373 MG glioma cell line. Third, SP induced new DNA synthesis and enhanced the proliferation rate of $NK_1R^+$, but not of $NK_1R^-$ glioma cell lines. Also, NKA stimulated the proliferation and cytokine secretion in $NK_1R^+$ glioma cell lines. All the stimulant effects of SP/NKA on $NK_1R^+$ glioma cell lines were completely blocked by a specific tachykinin $NK_1R$ antagonist, MEN 11467. These data support the potential use of tachykinin $NK_1R$ antagonist for controlling the proliferative rate of human gliomas.

**Keywords:** glioma; substance P; tachykinin $NK_1$ receptor; cytokine production; proliferation

Substance P (SP), an undecapeptide of the tachykinin family of peptides, is the preferential endogenous ligand for the tachykinin $NK_1$ receptor ($NK_1R$) (Maggi et al, 1993; Otsuka and Toshioka, 1993).

SP activates phospholipase C and stimulates the release of interleukin 6 (IL-6) and prostaglandin $E_2$ from human fetal astrocytes in culture (Palma et al, 1997), indicating an involvement of this neuropeptide in modulating astrocyte functions. Although there is little evidence for the expression of tachykinin $NK_1R$ by astrocytes in the normal adult brain (Maggi, 1997 for review), there is evidence for up-regulation of this receptor by reactive astrocytes: proliferating glial cells express high concentrations of $NK_1Rs$ after transection of the optic nerve (Mantyh et al, 1989). Moreover, SP-immunoreactive astrocytes were observed in multiple sclerosis plaques (Kostyk et al, 1989) and in the forebrains of human infants (Michel et al, 1986). Interestingly, high expression of SP receptors (Henning et al, 1995), as well as the presence of the SP itself, (Allen et al, 1985) has been described in human malignant gliomas such as astrocytomas and glioblastomas. These observations suggest a possible role of tachykinin $NK_1Rs$ in supporting the development and growth of human astrocytomas.

The human astrocytoma cell line U373 MG expresses high levels of tachykinin $NK_1R$ (Heuillet et al, 1993), and responds to applied SP by releasing taurine (Lee et al, 1992) and a panel of cytokines including IL-6, IL-8, leukaemia inhibitor factor (LIF) and granulocyte–macrophage colony-stimulating factor (GM-CSF) (Gitter et al, 1994; Palma et al, 1994, 1995; Palma and Manzini, 1998). The release of cytokines by malignant glioma cells has been associated with glioma progression (Gillespie, 1996); for example, IL-6 has been detected in tumour cysts and cerebrospinal fluids in patients harbouring malignant gliomas (Weller et al, 1991; Frei et al, 1992).

In addition to modulating cytokine production, SP induces DNA synthesis and activates the mitogen-activated protein (MAP) kinase pathway in U373 MG cells (Luo et al, 1996). However, the actual ability of this neuropeptide to produce proliferation of human glioma cells has not been determined until now.

In this study, we have further investigated the possible role of the tachykinin $NK_1R$ in modulating growth/development of human astrocytomas by addressing: (a) the expression of high-affinity tachykinin $NK_1R$ in different human glioma cell lines; (b) the presence/absence of functional responses (IL-6 secretion, DNA synthesis) to applied tachykinins in glioma cells lines positive or negative for expression of the tachykinin $NK_1R$ ($NK_1R^+$ and $NK_1R^-$ respectively); (c) the ability of SP to induce not only DNA synthesis but also actual proliferation of human glioma cells; (d) whether applied tachykinins induce the secretion of immunosuppressive cytokines (IL-10 and TGF-β1) from human gliomas; (e) the possibility that neurokinin A (NKA), which is often co-expressed with SP in the central nervous system, may affect glioma cells in the same way as SP does; and (f) the effect of a potent and selective antagonist for tachykinin $NK_1R$ on the responses of human glioma cells to SP.

*Received 9 April 1998*
*Revised 13 July 1998*
*Accepted 15 July 1998*

*Correspondence to:* C Palma, Menarini Ricerche, Department of Pharmacology, Via Tito Speri 10, 00040 Pomezia, RM, Italy

### MATERIALS AND METHODS

### Cell culture and media

Human glioma cell lines, U373 MG (astrocytoma grade III), SNB-19 (malignant glioblastoma), DBTRG-05 MG (glioblastoma multiforme), U138 MG (glioblastoma) were obtained from the

**Table 1** Inhibitory effect of the tachykinin $NK_1$ receptor antagonist MEN 11467 on SP and NKA-induced mitogenic effect on human glioma cell lines

| Conditions | SNB-19 | DBTRG-05 MG | U373 MG |
|---|---|---|---|
| Medium[a] | 58 000 ± 4999[b] | 27 147 ± 2936 | 55 333 ± 8671 |
| MEN 11467 (100 nM) | 60 000 ± 3945 | 28 586 ± 3789 | 58 000 ± 4999 |
| SP (100 nM) | 90 333 ± 5696** | 44 560 ± 3267* | 102 666 ± 8346** |
| SP (100 nM) + MEN 11467 (100 nM) | 60 666 ± 4055 | 29 706 ± 3336 | 61 666 ± 4055 |
| NKA (100 nM) | 97 333 ± 3179** | n.t. | 111 333 ± 8830** |
| NKA (100 nM) + MEN 11467 (100 nM) | 62 666 ± 4115 | n.t. | 60 666 ± 7115 |

[a]Human glioma cell lines, SNB-19, DBTRG-05 MG and U373 MG, were plated at 20 000 cells per well in appropriate medium containing 0.5%, 0.2% and 0.8% FBS respectively. Tachykinins and MEN 11467 were then added to the culture. After 72 h of incubation for DBTRG-05 MG and SNB-19 and 96 h for U373 MG, the cell numbers in the well were counted. [b]Mean of cell numbers ± s.e.m. of a triplicate. *$P < 0.05$ Anova one-way and Tukey test vs medium. **$P < 0.01$ Anova one-way and Tukey test vs medium. n.t., not tested.

American Type Culture Collection (Rockville, MD, USA); MOG-G-CCM (anaplastic astrocytoma) were purchased from ECACC (Salisbury, Wiltshire, UK). Cells were maintained in the appropriate media [U373 MG in RPMI-1640; SNB-19 in Ham's F10; U138 MG in Eagle's modified Eagle medium (MEM) with 0.1 mM non-essential amino acids, 1 mM sodium pyruvate and Earle's balanced salt solution (BSS); MOG-G-CCM in Ham's F10/ Dulbecco's modified Eagle medium (DMEM) (1:1); DBTRG-05 MG in RPMI-1640 with 10 mg $ml^{-1}$ of adenine (Sigma, St Louis, MO, USA), 1 mg $ml^{-1}$ of adenosine triphosphate (Sigma), 100 mg $ml^{-1}$ of L-cystine, 15 mg $ml^{-1}$ of hypoxanthine, 50 mg $ml^{-1}$ of L-isoleucine, 50 mg $ml^{-1}$ L-proline, 100 mg $ml^{-1}$ sodium pyruvate and 1 mg $ml^{-1}$ of thymidine] containing 10% heat-inactivated (65°C, 30 min) fetal bovine serum (FBS) with 5 mM HEPES, 2 mM L-glutamine, 100 U $ml^{-1}$ of penicillin and 100 µ $ml^{-1}$ of streptomycin. All reagents, except those otherwise indicated, were purchased from Gibco, Grand Island, NY, USA.

### Reagents

Substance P, septide and NKA were purchased from Calbiochem-Novabiochem, Laufelingen, Switzerland; lipopolysaccharide (LPS) from *Escherichia coli* 0111:B4 were obtained from Sigma; human recombinant IL-1β (specific activity $1.3 \times 10^7$ U $mg^{-1}$) was purchased from Janssen Biochimica, Beerse, Belgium. The compound MEN 11467 [indolyl-3-carboxy-*cis* g2 Ac6c-D-2-Nal-NMe-CO-$CH_2$(4-$CH_3$)phenyl] was synthesized in the Department of Chemistry of Menarini Ricerche, Pomezia, Italy. MEN 11467 is a potent, selective and specific ligand of human tachykinin $NK_1R$.

In binding studies, MEN 11467 showed high affinity for $NK_1Rs$ expressed in human cell lines ($K_i = 0.4 \pm 0.1$ and $K_i = 1.34 \pm 0.32$ for IM-9 and U373 MG respectively) and virtually no affinity ($K_i \geq 10\,000$ nM) for the $NK_1R$ present in rat urinary bladder membranes, a result more than 1000-fold selective for the human compared with the murine receptors. In addition, MEN 11467 has negligible effects on the binding of [$^{125}$I]NKA to hamster urinary bladder membranes ($NK_2$ receptor) or of [$^3$H]senktide to guinea pig cerebral cortex membranes ($NK_3$ receptor) (Cirillo et al, 1998).

### Binding assays

Binding assays were conducted on confluent intact cells in 24-well plastic culture dishes, as described previously (Goso et al, 1994) with minor modifications. Briefly, cells were rinsed and 500 µl of RPMI-1640 supplemented with 0.2% glucose and 1% bovine serum albumin was added to each well for 30 min. The buffer was then aspirated and fresh buffer containing [$^3$H]SP (specific activity 40 Ci $mmol^{-1}$; Amersham, Buckinghamshire, UK) or [$^3$H][$Sar^9$,Met($O_2$)$^{11}$]SP (specific activity 40.6 Ci $mmol^{-1}$; New England Nuclear, Du Pont de Nemours, NEN Division, Dreiech, Germany) was added in a final volume of 500 µl. The non-specific binding was defined as that displaceable by unlabelled 10 µM SP or [$Sar^9$,Met($O_2$)$^{11}$]SP. The plates were incubated for 2 h at 4°C. The reaction was stopped by aspirating the medium and then rinsing each well three times with 1 ml of 0.9% sodium chloride in 10 mM HEPES (pH 7.2) wash buffer. The cells were solubilized in 5% sodium dodecyl sulphate in 0.01 M hydrochloride acid (0.5 ml) at 37°C for 1 h and radioactivity was quantified in a liquid scintillation spectrometer.

For saturation binding experiments, cells were incubated with increasing concentrations of [$^3$H]SP (0.1–5 nM) or [$^3$H][$Sar^9$,Met($O_2$)$^{11}$]SP (0.1–5 nM). For competition binding experiments, cells were incubated with 2 nM of both radioligands, in the presence of varying amounts of competitor. Binding data were analysed using the iterative curve fitting program, Ligand (Munson and Rodbard, 1980).

### DNA synthesis

Human glioma cells were plated on 24-well tissue culture plates ($2 \times 10^4$ cells per well in 1 ml of medium, unless otherwise noted) in their appropriate media with low concentration or without FBS as indicated throughout the text. The stimulants were then added and left in the culture media for the entire length of the experiment. [$^3$H]methyl-thymidine [specific activity 82.5 Ci $mmol^{-1}$, NEN-Du Pont De Nemours Italiana, Cologno Monzese (MI), Italy] at a concentration of 1 µCi $ml^{-1}$ was added to each cell culture for the last 24 h of incubation. At the end of each experimental point, the cells were treated with 1 ml of 5% cold trichloroacetic acid (TCA) for 10 min at 4°C. The acid was removed, and the cells were washed with double-distilled water (three times, 2 ml each time). The cells were removed from each well with 0.5 ml of 0.1 N sodium hydroxide and incubated for 15 min. Each sample was then neutralized with 0.1 N hydrochloric acid and the radioactivity was quantified in a liquid scintillation spectrometer.

### Proliferation studies

Human glioma cells were plated on 24-well tissue culture plates ($2 \times 10^4$ cells per well in 1 ml of medium, unless otherwise noted)

© Cancer Research Campaign 1999

in their appropriate media in serum-free conditions or with low-concentration FBS as indicated. The stimulants were then added and left in the culture medium for the entire experiment length. At the end of each experimental point, the medium was aspirated and the living cells detached with 200 µl of trypsin. The cells were then diluted in the vital colorant trypan blue (1:1) and counted in haemocytometer counting chambers (Newbauer and Nageotte).

### Detection of IL-6, IL-10 and TGF-β1 in supernatants of human glioma cell lines

Glioma cells were detached by trypsinization, washed in serum-free RPMI-1640, seeded in their appropriate FBS medium at $3.5\times10^5$ cell per well in 24-well culture plates, and allowed to adhere for 24 h at 37°C. Then the cultured medium was removed, the cells were washed three times in serum-free RPMI-1640, and fresh media containing low (1%) or high (10%) FBS were added. Cells were cultured for 18 h, unless otherwise indicated, in the presence or absence of SP, NKA, IL-1β. When necessary, the $NK_1$ receptor antagonists were co-administered with the stimulants. In all experimental conditions, cell viability was always greater than 99%, as determined by the Trypan Blue exclusion method. After the incubation time, the supernatants were collected and spun free of cells and debris. IL-6, IL-10 and TGF-β1 levels were assayed by specific ELISA kits (Quantikine, Research and Diagnostic System, Minneapolis, MN, USA or Biotrak, Amersham). To activate latent TGF-β1 to the immunoreactive form, 0.5 ml of cell culture supernatants were incubated for 10 min at room temperature with 0.1 ml 1 N hydrochloric acid. The acidified samples were then neutralized by adding 0.1 ml 1.2 N sodium hydroxide/0.5 M HEPES before being assayed. Significant levels of latent TGF-β1 were found in FBS, whereas no IL-6 or IL-10 was detected in FBS media even when FBS was present at 10%.

The data were analysed using linear regression of the Microplate Manager Program (Bio-Rad Laboratories, Richmond, CA, USA), and simultaneous fitting of families of sigmoidal dose-response curves using the four-parameter logistic equation with statistical analysis and elaboration of $IC_{50}$ and $EC_{50}$ values was performed using the program Allfit version 1.05 (De Lean et al, 1978).

### Statistical analysis

All data in the text and figures are means ± s.e.m. Statistical analysis of data was performed with Anova one-way and Tukey tests.

### RESULTS

### Identification and characterization of SP binding sites ($NK_1R$) on human glioma cell lines

By studying the specific binding of [$^3$H]SP, evidence for expression of tachykinin $NK_1R$ was obtained for SNB-19 and DBTRG-05 MG cell lines in addition to U373 MG cells. In these cell lines, the specific binding of [$^3$H]SP was usually >80% of total binding, as defined in the presence of 10 µM unlabelled SP. The binding of [$^3$H]SP was saturable and Scatchard analysis indicated a single population of high-affinity binding sites: the $K_d$ and $B_{max}$ values obtained in the three cell lines are reported in Figure 1 along with the corresponding Scatchard plots.

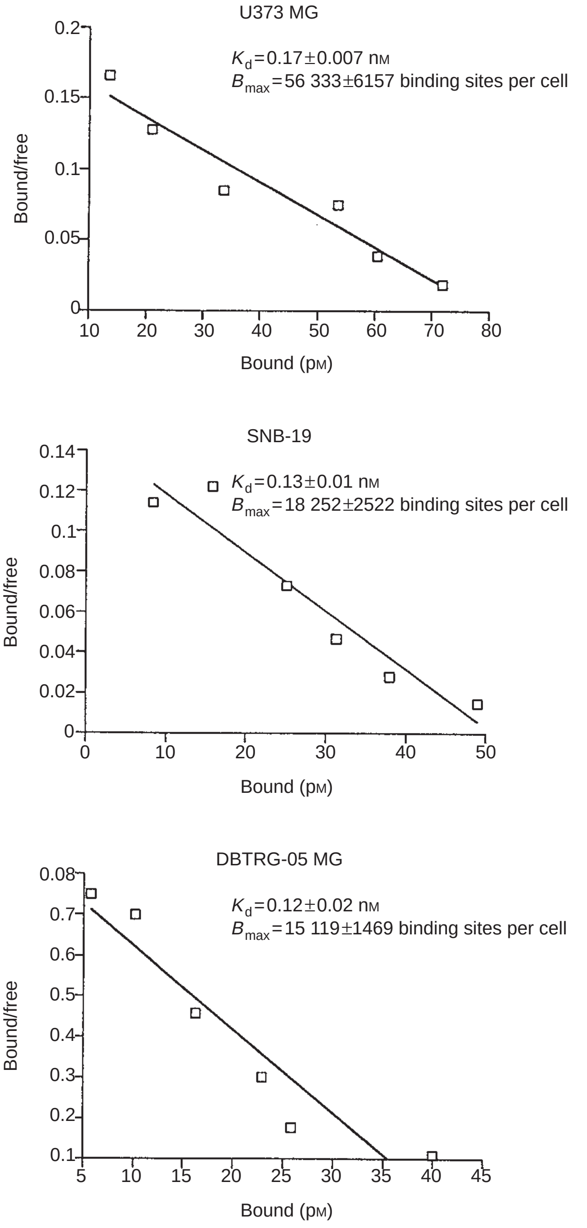

**Figure 1** Scatchard analysis of specific [$^3$H]SP binding to human glioma cell lines. Data for Scatchard plots are from one experiment representative of three run in triplicate, whereas the indicated $K_d$ and $B_{max}$ values are from the simultaneous analysis with Ligand program of the three independent experiments

Because the presence of tachykinin $NK_1R$ in SNB-19 and DBTRG-05 MG cell lines was not studied previously, saturation binding assays with the synthetic $NK_1R$ selective ligand [$^3$H][Sar$^9$,Met(O$_2$)$^{11}$]SP were also performed in these cell lines. Scatchard analysis showed again a single population of high-affinity binding sites ($K_d$ of 0.12 ± 0.04 nM and $B_{max}$ of 22 062 ± 3309 for SNB-19 cells; $K_d$ of 0.08 ± 0.02 nM and $B_{max}$ of 13 664 ± 1370 for DBTRG-05 MG cells).

Specific binding of [$^3$H]SP or [$^3$H][Sar$^9$,Met(O$_2$)$^{11}$]SP was not observed in U138 MG and MOG-G-CCM cells, indicating that these human glioma cell lines do not express typical $NK_1R$.

© Cancer Research Campaign 1999

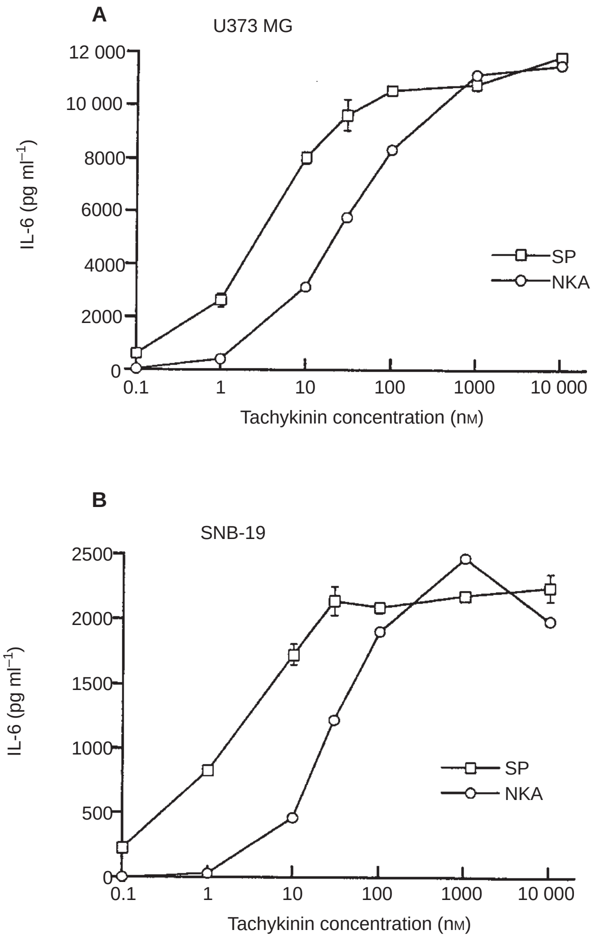

**Figure 2** Release of IL-6 induced by SP and NKA in human glioma cell lines. U373 MG and SNB-19 cells were cultured for 18 h in FBS 1% medium in the presence of SP or NKA at the indicated concentrations before collection of supernatants for IL-6 measurement with a specific ELISA kit. The data are presented as means±s.d. of triplicate determinations after subtraction of spontaneous IL-6 secretion (250±15 and 900±13 pg ml$^{-1}$ for U373 MG and SNB-19 cells respectively) of one representative experiment

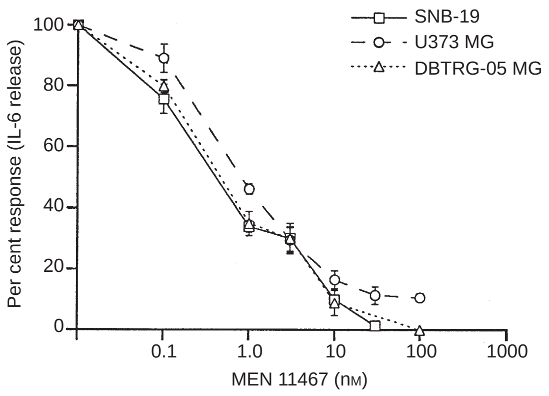

**Figure 3** Inhibition of SP-induced IL-6 release in human glioma cell lines by the tachykinin NK$_1$ receptor antagonist MEN 11467. U373 MG, SNB-19 and DBTRG-05 MG cells, cultured in FBS 1% medium, were treated with SP (100 nM) (in combination with IL-1β 100 U ml$^{-1}$ for DBTRG 05 MG cells) in the absence or presence of increasing concentrations of MEN 11467 for 18 h. IL-6 protein levels were determined in culture supernatants by specific ELISA. The data are presented as per cent of inhibition vs the amount of IL-6 released in absence of MEN 11467; the data are the means±s.e.m. of four independent experiments for U373 MG cells and the means±s.e.m. of triplicate determinations of one representative experiment for SNB-19 and DBTRG-05 MG cells

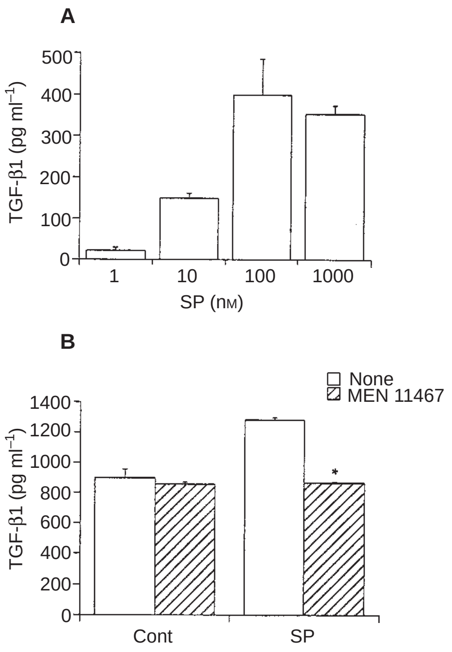

**Figure 4** Release of TGF-β1 induced by SP in U373 MG cells. U373 MG were treated with increasing concentration of SP (**A**) or with SP (100 nM) in the absence or presence of MEN 11467 (100 nM) (**B**) for 18 h. TGF-β1 protein levels were determined in culture supernatants, previous activation of the latent form, by specific ELISA. Values are means±s.e.m. of three independent experiments run in duplicate (**A**) or means±s.e.m. of triplicate determinations of one representative experiment (**B**). *$P < 0.01$ Anova one-way and Tukey test vs the condition in absence of MEN 11467

### SP and NKA stimulate the release of IL-6 from NK1R$^+$ glioma cell lines

SNB-19 and U373 MG glioma cell lines are able to spontaneously release IL-6, not only in medium containing high-concentration FBS (10%) (Gitter et al, 1994; Palma et al, 1994, 1995; Palma and Manzini, 1998) but also when cultured in low-concentration FBS (1%) (Figure 2). Even in the more stringent culture conditions, SP and NKA induced a concentration-dependent release of IL-6. The $EC_{50}$ values were 2 ± 0.4 and 3.8 ± 0.5 nM for SP in SNB-19 and U373 MG glioma cells, respectively; the corresponding $EC_{50}$ values for NKA were about tenfold higher than for SP (37 ± 9 and 26 ± 7 nM for U373 MG and SNB-19 cells, respectively).

DBTRG-05 MG cells, which do not spontaneously secrete IL-6, did not respond with augmented IL-6 secretion in response to added SP ($n$ = 3). However, if challenged with IL-1β (100 U ml$^{-1}$), a secretion of IL-6 was established (686 ± 20 pg ml$^{-1}$) from DBTRG-05 MG cells; in these conditions, SP produced a concentration-dependent potentiation of the response to IL-1β (739 ± 2, 893 ± 22, 1105 ± 26 and 1186 ± 15 pg ml$^{-1}$ at 1, 10, 100 and 1000 nM of SP, respectively).

The involvement of the tachykinin NK$_1$R in the stimulant action of SP on basal or stimulated (in DBTRG-05 MG cells) secretion of IL-6 was confirmed by studying the inhibitory action of the potent and selective tachykinin NK$_1$R antagonist MEN 11467 (Figure 3). MEN 11467 produced a concentration-dependent and comparable inhibition of the responses to SP in the three NK$_1$R$^+$ glioma cell lines

© Cancer Research Campaign 1999

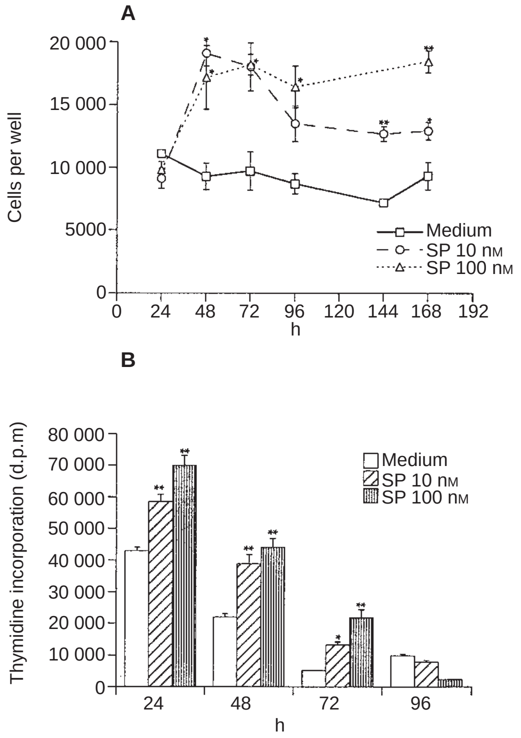

**Figure 5** SP effects on U373 MG cellular growth. U373 MG cells (20 000 cells per well) were stimulated in serum-free medium with SP (10 or 100 nM), and at the indicated time points the number of cells in the well were counted by haemocytometer counting chamber (**A**) or [$^3$H]methyl-thymidine incorporation into DNA was measured (**B**). [$^3$H]methyl-thymidine was added to the cell culture for the last 24 h of incubation. Data are presented as means±s.e.m. of triplicate determination of one representative experiment. *$P < 0.05$ Anova one-way and Tukey test vs medium. **$P < 0.01$ Anova one-way and Tukey test vs medium with $IC_{50}$ values of 0.8 ± 0.1, 0.6 ± 0.2 and 0.6 ± 0.3 nM for U373 MG, SNB-19 and DBTRG-05 MG cells, respectively (Figure 3).

On its own, MEN 11467 neither significantly affected the basal secretion of IL-6 from SNB-19 and U373 MG cell lines (486 ± 26 and 420 ± 49 pg ml$^{-1}$; 900 ± 16 and 843 ± 5 pg ml$^{-1}$ in the absence or presence of 100 nM MEN 11467 for SNB-19 and U373 MG cells respectively), nor the IL-6 secretion induced by IL-1β from DBTRG-05 MG cells (690 ± 17 pg ml$^{-1}$ and 604 ± 11 pg ml$^{-1}$ in the absence or presence of 100 nM MEN 11467, respectively).

To confirm the specificity of the stimulatory effects of SP, the effect of this peptide was also investigated in $NK_1R^-$ glioma cell lines; up to 100 nM, SP neither induced IL-6 secretion nor affected IL-1β-stimulated IL-6 release from U138 MG or MOG-G-CCM cells (data not shown, $n = 2$ for each cell line).

### SP stimulates the production of TGF-β1 but not IL-10 from U373 MG cells

The possible secretion of immunodepressant cytokines, IL-10 and TGF-β1, was investigated in U373 MG cells. No spontaneous secretion of IL-10 was measured nor any secretory response was observed upon challenge with IL-1β (100 U ml$^{-1}$), LPS (1–20 ng ml$^{-1}$) and SP (1–1000 nM) for 24–72 h in the presence of low- (1%) or high-concentration (10%) FBS ($n = 3$).

In contrast, a spontaneous release of the inactive form of TGF-β1 was detected (1033 ± 92 pg ml$^{-1}$) after 24 h of culture in the

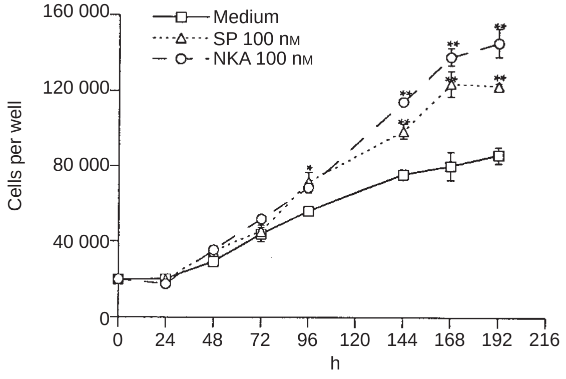

**Figure 6** Tachykinin effects on U373 MG cellular growth in the presence of serum. U373 MG cells (20 000 cells per well) were stimulated in medium containing FBS at 0.8% with SP or NKA (100 nM). At the indicated time points, the number of cells in the well were counted by haemocytometer counting chamber. Data are presented as means±s.e.m. of triplicate determination of one representative experiment. *$P < 0.05$ Anova one-way and Tukey test vs medium. **$P < 0.01$ Anova one-way and Tukey test vs medium

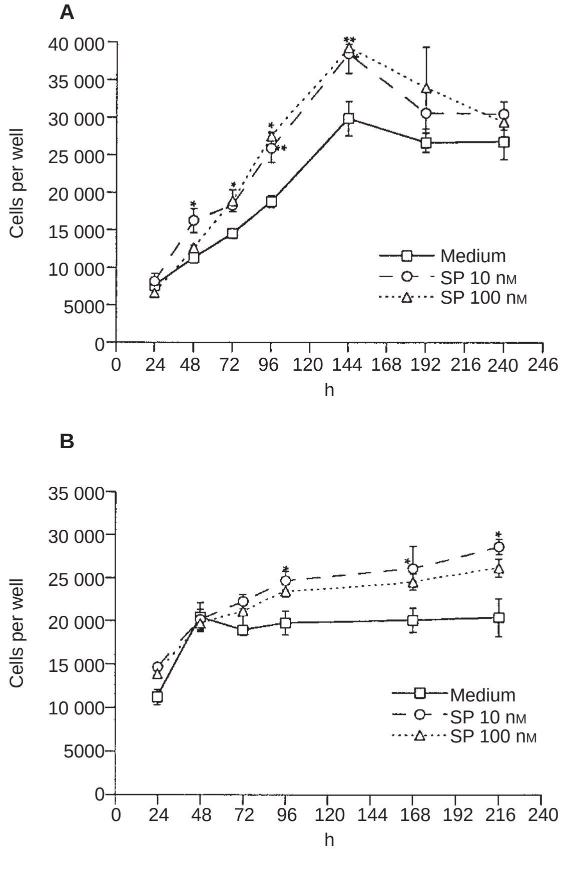

**Figure 7** SP effects on SNB-19 and DBTRG-05 MG cellular growth. SNB-19 cells (**A**) (20 000 cells per well) in serum-free medium or DBTRG-05 MG cells (**B**) (20 000 cells per well) in medium containing FBS at 0.2% were stimulated with SP (10 or 100 nM). At the indicated time points, the number of cells in the well were counted by haemocytometer counting chamber. Data are presented as means±s.e.m. of triplicate determination of one representative experiment. *$P < 0.05$ Anova one-way and Tukey test vs medium. **$P < 0.01$ Anova one-way and Tukey test vs medium

 *© Cancer Research Campaign 1999*

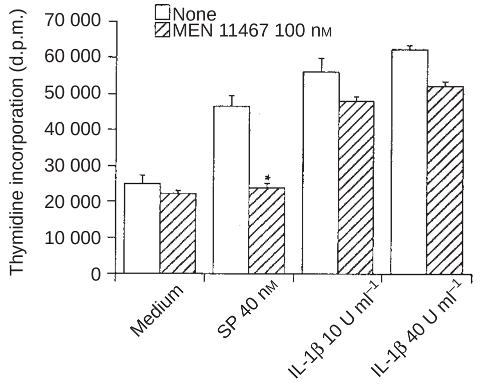

**Figure 8** Inhibition of SP-induced DNA synthesis in U373 MG cells by the tachykinin $NK_1$ receptor antagonist MEN 11467. U373 MG cells (20 000 cells per well) were stimulated in serum-free medium containing 1 µCi $ml^{-1}$ of [$^3$H]methyl-thymidine with SP (40 nM) or IL-1β (10 or 40 U $ml^{-1}$) in the absence or presence of MEN 11467 (100 nM) for 24 h. TCA-precipitable material was quantified by liquid scintillation counting and plotted as [$^3$H]methyl-thymidine incorporation. Data are presented as means±s.e.m. of triplicate determination of one representative experiments. *$P < 0.01$ Anova one-way and Tukey test vs the condition in absence of MEN 11467

presence of low-concentration FBS (1%). SP potentiated, in a concentration-dependent manner, the secretion of the inactive form of TGF-β1 (Figure 4A). This response involves the activation of tachykinin $NK_1R$ because MEN 11467 (100 nM) completely blocked the SP (100 nM)-stimulated release of TGF-β1 (Figure 4B). However, the presence of active TGF-β1 molecules was never observed in the supernatants of SP-triggered U373 MG cells. TGF-β1 release was not modulated by stimulation with LPS (20 ng $ml^{-1}$) or IL-1β (100 U $ml^{-1}$).

### SP induces DNA synthesis and proliferation in $NK_1R^+$ glioma cell lines

The aim of these experiments was to verify the ability of SP to stimulate growth of human glioma cell lines by measuring both DNA synthesis (assessed by evaluating thymidine uptake) and cell proliferation (by counting the number of cells at different times from exposure to SP).

In a first series of experiments (Figure 5), U373 MG cells were plated at low concentration ($2\times10^4$ cells per well in 24-well tissue culture plates) in serum-free medium, and time course experiments were performed in the presence of SP. No significant increase in the number of cells was observed in control conditions (Figure 5); SP (10–100 nM) increased DNA synthesis already after 24 h of stimulation, whereas the number of cells present in the wells at this time was similar for treated and untreated samples. However, the addition of SP determined a doubling of cell number after 48 h of culture (Figure 5).

In another series of experiments (Figure 6), the ability of SP to stimulate the growth of U373 MG cells was determined in the presence of low-concentration serum (0.8%), which stimulates cell growth to some extent; as shown in Figure 6, a stimulant action of SP on cell number above the spontaneous growth was observed since 96 h from addition of the neuropeptide and a stimulant effect was evident up to 192 h (Figure 6). In these conditions, the mitogenic effect of SP was not concentration-dependent (1–1000 nM); the proliferative response observed at 1 nM was similar to that obtained at the highest concentration (55 333 ± 8671, 94 000 ± 11 222 and 102 000 ± 6060 cells per well after 96 h of incubation in medium alone, SP 1 nM and SP 1000 nM, respectively).

A third series of experiments showed (Figure 7) that application of SP determines a mitogenic effect on other $NK_1R^+$ human glioma cell lines, SNB-19 and DBTRG-05 MG cell lines. In contrast, the $NK_1R^-$ cell lines U138 MG and MOG-G-CCM did not show any increase in DNA synthesis (after 48 h of stimulation, data not shown, $n = 2$ for each cell line) or proliferation after challenge with SP (55 000 ± 4582 and 61 666 ± 7023 cells per well; 81 000 ± 11 357 and 77 000 ± 4582 cells per well after 96 h of incubation in absence or in the presence of 100 nM SP for U138 MG and MOG-G-CCM, respectively).

### An $NK_1R$ antagonist inhibits SP-induced growth in glioma cell lines

The tachykinin $NK_1R$ antagonist MEN 11467 (100 nM) completely reverted the SP-induced thymidine uptake by U373 MG cells, whereas that induced by IL-1β (10 and 40 U $ml^{-1}$) was not significantly reduced (Figure 8). Moreover, the enhanced proliferation of $NK_1R^+$ glioma cell lines induced by SP was inhibited by MEN 11467 (Table 1).

### NKA stimulates glioma cell growth via tachykinin $NK_1Rs$

Like SP (Figure 5), NKA increased the thymidine uptake in U373 MG cells (38 476 ± 870, 56 855 ± 2794 and 71 827 ± 3949 d.p.m., after 24 h culture in serum-free medium, NKA 10 nM and NKA 100 nM, respectively). Moreover, in experiments performed in media containing low-concentration serum (0.8%), NKA (100 nM) stimulated the proliferation of SNB-19 cell and U373 MG cell lines (Figure 7 and Table 1). The mitogenic effect of NKA was completely blocked by MEN 11467 (Table 1).

### DISCUSSION

The present findings expand previous observations (Luo et al, 1996; Sharif et al, 1996) on the role played by tachykinins (via $NK_1R$) in producing biological responses which are potentially relevant for the development and growth of human gliomas. In particular, the present study provides the following novel observations: (a) in addition to U373 MG cells, two glioma cell lines have been characterized as being $NK_1R^+$ (SNB-19 and DBTRG-05 MG), whereas two other cell lines were found to be $NK_1R^-$ (MOG-G-CCM and U138 MG); (b) the demonstration of the mitogenic properties of SP in terms not only of DNA synthesis but also as an actual increase in glioma cell number; (c) a positive correlation has been established between functional responses (modulation of IL-6 secretion and proliferation) of human glioma cell lines and the expression of $NK_1R$; (d) in keeping with the above, the activation of human glioma cell lines by tachykinins was blocked by the highly potent and selective tachykinin $NK_1R$ antagonist MEN 11467; (e) in addition to SP, NKA also stimulates cytokine secretion and enhances proliferation in human glioma cell lines via the $NK_1R$; (f) stimulation of the $NK_1R$ also determines the release of the immunosuppressant cytokine TGF-β1 from the U373 MG cell line.

Application of SP or of tachykinin $NK_1R$ selective agonists determines a number of functional responses in U373 MG glioma cells: the stimulation of phosphatidyl inositol (Pi) turnover,

© Cancer Research Campaign 1999

mobilization of intracellular calcium, release of taurine, activation of MAP kinase and secretion of cytokines (Eistetter et al, 1992; Lee et al, 1992; Gitter et al, 1994; Palma et al, 1994, 1995; Luo et al, 1996; Palma and Manzini, 1998). Therefore, the activation of $NK_1R$ initiates a set of cellular responses which eventually includes stimulation of DNA synthesis, cell division and proliferation. The actual proliferation of human glioma cells detected in this study occurs both in serum-free conditions and in the presence of serum; the latter observation (Figure 6) indicates that SP can co-operate with other growth factors in regulating the proliferation of $NK_1R^+$ cells. In contrast, the former observation indicates that SP is in itself a sufficient stimulus for promoting glioma cell proliferation. Even in the most stringent serum-free conditions, an actual proliferative response to SP was evident only at > 24 h from its applications; therefore, the question arises as to whether the continuous occupancy of $NK_1R$ during this time lag is required for the triggering of the proliferative response, or, as an alternative, whether the initial stimulus provided by SP determines glioma cell proliferations indirectly through the release of other factors (e.g. IL-6, see below). As a matter of fact, SP application induces a lasting expression of κB-dependent genes (Lieb et al, 1998), such as expression of mRNA coding for the mitogenic cytokine IL-6 (Palma and Manzini, 1998). It appears, therefore, conceivable that the actual proliferation of glioma cells, as detected in this study, had involved some intermediate step in the sequence of events initiated by the occupancy of tachykinin $NK_1R$. The secretion of cytokines such as IL-6 or TGF-β1 could provide an autocrine /paracrine loop for glioma progression. In fact, both IL-6 and TGF-β1 roles facilitate glioma cell proliferation (Jennings et al, 1991; Munoz-Fernandes and Fresno, 1993).

At the present time, we cannot totally exclude that, in addition to its role as growth promoter, SP may also have enhanced glioma cell survival and plating efficiency. However, the initial number of cells present in the wells was similar in the presence or absence of SP, suggesting that a putative effect of this neuropeptide on plating efficiency, if any, was a minor one. In contrast, further studies should be performed to clarify whether SP can increase the number of cells entering the cell cycle and/or induce an improved survival by preventing apoptosis, which is a common event in tumour cells lacking in growth factors.

The present findings demonstrate that, in addition to SP, NKA also stimulates the growth of human glioma cells via the $NK_1R$. This observation is of particular interest because SP and NKA can be produced through the expression of the same gene in the CNS and are frequently co-localized and co-released by the same neurons (Maggi et al, 1993; Otsuka and Yoshioka, 1993 for reviews). NKA is the natural ligand with preferential affinity for tachykinin $NK_2$ receptors, but it can also act as a full agonist at $NK_1Rs$ (Maggi and Schwartz, 1997 for review). Indeed, because $NK_2$ receptors are poorly expressed, if not absent, in the mammalian CNS (Otsuka and Yoshioka, 1993), it is currently believed that SP and NKA act in concert by stimulating $NK_1R$ at most mammalian CNS synapses.

Sharif et al (1996) reported that activation of the MAP kinase pathway and [$^3$H]thymidine incorporation into DNA were induced by NKA as efficiently as SP when fixed high equimolar concentrations of tachykinin were used (100 nM and 1 μM respectively). In our hands, NKA induced responses (secretion of IL-6, stimulation of DNA synthesis and cell proliferation) which were quantitatively comparable to those produced by SP. However, our data document that NKA has about a ten fold lower potency in stimulating IL-6 secretion from $NK_1R^+$ human glioma cell lines than SP, in accordance with the lower affinity of NKA for $NK_1Rs$, as shown in competition binding vs [$^3$H]SP in U373 MG cells (Heuillet et al, 1993) and in SNB-19 cells (our unpublished observation). Our findings provide unequivocal evidence that the actions of NKA on human gliomas are mediated via $NK_1R$ stimulation. In fact NKA was active in $NK_1R^+$ but not in $NK_1R^-$ cell lines, and its effects in $NK_1R^+$ glioma cells were blocked by MEN 11467.

We also demonstrated that SP, via $NK_1R$ occupancy, stimulates the secretion of the immunodepressant cytokine TGF-β1. This finding is of particular interest because it provides an additional mechanism through which tachykinins may facilitate the growth and development of human gliomas. TGF-β1 and IL-6 contribute to the general immunodepression observed in glioma patients (Roszman et al, 1991; Ausiello et al, 1991). IL-6 inhibits the secretion of IL-1β and TNF-α and can counteract the activation of the immune system in this way (Schindler et al, 1990). TGF-β1 induces apoptosis of tumour-infiltrating lymphocytes, significantly reducing their cytotoxic properties, and depresses the activity of natural killer cells (Bodmer et al, 1989; Weller et al, 1995). Therefore, a stimulation by tachykinins of the local release of immunosuppressant cytokines could play a facilitatory role in the growth and development of gliomas by helping tumour cells to evade attack by the immune system.

High-grade human malignant glioma are inevitably lethal neoplasms, and the median survival of patients treated with standard cytoreductive surgery and post-operative radiotherapy is in the range of 1 year. The task of developing additional or novel treatments crucially requires an increase in knowledge of the physiology and molecular biology of these brain tumours. We have gathered evidence indicating that tachykinins (SP and NKA), through a specific stimulation of $NK_1R$, have functional effects on several human glioma cell lines, resulting in proliferation, mitogenesis and release of soluble factors which can influence the tumour cell–host interactions including modulation of the immune system.

Altogether, these observations suggest a possible role of tachykinins, via $NK_1Rs$ in glioma development and growth. It should be noted that SP was found in human glioma tissues (Allen et al, 1985), and human astrocytes themselves are a possible source of tachykinins (Michel et al, 1986). In addition, an overexpression of $NK_1Rs$ seems to occur in gliomas in relation to their degree of malignancy (Henning et al, 1995). Therefore, an autocrine/paracrine loop providing a facilitatory input on tumour growth could be devised to exist in the glioma itself. In addition, the expression of $NK_1Rs$ in peritumoral and tumoral blood vessels (Henning et al, 1995) suggests a role of SP in facilitating tumour blood supply by virtue of its $NK_1R$-mediated angiogenic and vasodilator properties (Ziche et al, 1990). Tachykinins are multifaceted factors for amplification of the malignant growth of human glioma cells. In view of the obligatory role of $NK_1Rs$ in all these effects of tachykinins, the pharmacological blockade of $NK_1Rs$ could represent a new strategy to slow down glioma tumour growth in humans.

### REFERENCES

Allen JM, Hoyle NR, Yeats JC, Ghatei MA, Thomas DG and Bloom SR (1985) Neuropeptides in neurological tumours. *J Neurooncol* **3**: 197–202

Ausiello CM, Palma C, Maleci A, Spagnoli GC, Amicis C, Antonelli G, Casciani CU and Cassone A (1991) Cell-mediated cytotoxicity in glioma patients: analysis of effector cells and lymphokines production induced by microbial antigen in peripheral blood mononuclear cells. *Eur J Cancer* **27**: 646–650

 © Cancer Research Campaign 1999

Bodmer S, Strommer K, Frei K, Siepl C, De Tribolet N, Heid I and Fontana A (1989) Immunosuppresion and transforming growth factor beta in glioblastoma. Preferential production of transforming growth factor-beta 2. *J Immunol* **143**: 3222–3229

Cirillo R, Astolfi M, Ciucci A, Palma C, Parlani M, Lopez G, Conte B, Terracciano R, Fincham CI, Sisto A, Maggi CA and Manzini S (1998) Pharmacology of MEN 11467, a potent, selective and orally effective pseudopeptide tachykinin NK-1 antagonist. *Naun Schmied Arch Pharmacol* **358** (Suppl 1): R324

De Lean A, Munson PJ, Guardabasso V and Rodbard D (1978) Simultaneous analysis of families of sigmoidal curves: application to bioassay, radioligand assay, and physiological dose–response curves. *Am J Physiol* **235**: E97–E102

Eistetter HR, Mills A, Brewster R, Alouani S, Rambosson C and Kawashima E (1992) Functional characterization of neurokinin-1 receptors on human U373 MG astrocytoma cells. *Glia* **6**: 89–95

Frei K, Piani D, Malipiero UV, Van Meir E, de Tribolet N and Fontana A (1992) Granulocyte–macrophage colony-stimulating factor (GM-CSF) production by glioblastoma cells. Despite the presence of inducing signals GM-CSF is not expressed in vivo. *J Immunol* **148**: 3140–3146

Gillespie GY (1996) Cytokines as modulators of malignant glioma progression. In *Cytokines and the CNS*. Ransohoff RM and Beneviste EM (eds.), pp. 269–286. CRC Press: Boca Raton

Gitter BD, Regoli D, Howbert JJ, Glasebrook AL and Waters DC (1994) Interleukin-6 secretion from human astrocytoma cells induced by substance P. *J Neuroimmunol* **51**: 101–108

Goso C, Potier E, Manzini S and Szallasi A (1994) Comparison of tachykinin NK1 receptors in human IM9 and U373 MG cells, using antagonist [FK888, (±)-CP-96,345 and RP 67580] binding. *Eur J Pharmacol* **254**: 221–227

Henning IM, Laissue JA, Horisberger U and Reubi JC (1995) Substance-P receptors in human primary neoplasms: tumoral and vascular localization. *Int J Cancer* **61**: 786–792

Heuillet E, Menager J, Faradin V, Flamand O, Bock M, Garret C, Crespo A, Fallourd AM and Doble A (1993) Characterization of a human NK1 tachykinin receptor in the astrocytoma cell line U373 MG. *J Neurochem* **60**: 868–876

Jennings MT, Maciunas RJ, Carver R, Bascom CC, Juneau P, Misulis K and Moses HL (1991) TGFβ1 and TGFβ2 are potential growth regulators for low-grade and malignant glioma in vitro: evidence in support of an autocrine hypothesis. *Int J Cancer* **49**: 129–139

Kostyk SK, Kowall NW and Hauser SL (1989) Substance P immunoreactive astrocytes are present in multiple sclerosis plaques. *Brain Res* **504**: 284–288

Lee CM, Tung WL and Young JD (1992) Tachykinin-stimulated inositol phospholipid hydrolysis and taurine release from human astrocytoma cells. *J Neurochem* **59**: 406–414

Lieb K, Fiebich BL, Berger M, Bauer J and Schulze-Osthoff K (1998) The neuropeptide substance P activates transcription factor NF-κB and κB-dependent gene expression in human astrocytoma cells. *J Immunol* **159**: 4952–4958

Luo W, Sharif TR and Sharif M (1996) Substance P-induced mitogenesis in human astrocytoma cells correlates with activation of the mitogen-activated protein kinase signaling pathway. *Cancer Res* **56**: 4983–4991

Maggi CA (1997) The effects of tachykinins on inflammatory and immune cells. *Regul Peptides* **70**: 75–90

Maggi CA and Schwartz TW (1997) The dual nature of the tachykinin $NK_1$ receptor. *Trends Pharmacol Sci* **18**: 351–354

Maggi CA, Patacchini R, Rovero P and Giachetti A (1993) Tachykinin receptors and tachykinin receptor antagonists. *J Auton Pharmacol* **13**: 23–93

Mantyh PW, Johnson DJ, Boehmer CG, Catton MD, Vinters HV, Maggio JE, Too HP and Vigna SR (1989) Substance P receptor binding sites are expressed by glia in vivo after neuronal injury. *Proc Natl Acad Sci USA* **86**: 5193–5197

Michel JP, Sakamoto N, Bouvier R, Tommasi M and Pearson J (1986) Substance P-immunoreactive astrocytes related to deep white matter and striatal blood vessels in human brain. *Brain Res* **377**: 383–387

Munoz-Fernandez MA and Fresno P (1993) Involvement of nitric oxide on the cytokine induced growth of glial cells. *Biochem Byophys Res Commun* **194**: 319–325

Munson PJ and Rodbard D (1980) A versatile computerized approach for the analysis of ligand binding data. *Anal Biochem* **107**: 220–239

Otsuka M and Toshioka K (1993) Neurotransmitter functions of mammalian tachykinins. *Physiol Rev* **73**: 229–308

Palma C and Manzini S (1998) Substance P induces secretion of immunomodulatory cytokines by human astrocytoma cells. *J Neuroimmunol* **81**: 127–137

Palma C, Goso C and Manzini S (1994) Different susceptibility to neurokinin-1 receptor antagonists of substance P and septide-induced interleukin-6 release from U373 MG human astrocytoma cell line. *Neurosci Lett* **171**: 221–224

Palma C, Urbani F and Manzini S (1995) Interleukin-6 production by U373 MG, a human astrocytoma cell line: different pathways involved in substance P and lipopolysaccharide activation. *J Neuroimmunol* **59**: 155–163

Palma C, Minghetti L, Astolfi M, Ambrosini E, Ceccherini Silberstein F, Manzini S, Levi G and Aloisi F (1997) Functional characterization of substance P receptors on cultured human spinal cord astrocytes: synergism of substance P with cytokines in inducing interleukin-6 and prostaglandin $E_2$ production. *Glia* **21**: 183–193

Roszman T, Elliott L and Brooks W (1991) Modulation of T-cell function by gliomas. *Immunol Today* **12**: 370–374

Schindler R, Mancilla J, Endres S, Ghorbani R, Clark SC and Dinarello CA (1990) Correlations and interactions in the production of interleukin-6 (IL-6), IL-1 and tumor necrosis factor (TNF) in human blood mononuclear cells: IL-6 suppress IL-1 and TNF. *Blood* **75**: 40–47

Sharif TR, Luo W, Houghton PJ and Sharif M (1996) Substance K peptide induces mitogenesis by activating the mitogen-activated protein kinase signaling pathway through the substance P receptor (NK-1 subtype) in human astrocytoma. *Cell Pharmacol* **3**: 441–449

Weller M, Stevens A, Sommer N, Melms A, Dichgans J and Wietholter H (1991) Comparative analysis of cytokine patterns in immunological, infectious and oncological neurological disorders. *J Neurol Sci* **104**: 215–221

Weller M, Malipiero U, Resing-Ehl A, Barr PJ and Fontana A (1995) Fas/APO-1 gene transfer for human malignant glioma. *Cancer Res* **55**: 2936–2944

Ziche M, Morbidelli L, Pacini M, Geppetti P, Alessandri G and Maggi CA (1990) Substance P stimulates neovascularisation in vivo and proliferation of cultured endothelial cells. *Microvasc Res* **40**: 264–278

© Cancer Research Campaign 1999
